# Willingness of Dutch general practitioners to grant euthanasia and assisted suicide requests: a comparative study of physical and mental health conditions

**DOI:** 10.1186/s12910-025-01333-y

**Published:** 2025-11-25

**Authors:** Esmee P. G. M. Jenniskens, Nils Mevenkamp

**Affiliations:** Department Nonprofit, Social & Health Care Management, Management Center Innsbruck, Universitaetsstrasse, Innsbruck, 15 6020 Austria

**Keywords:** Euthanasia, Assisted Suicide, General Practitioners, Mental Health Conditions, Physical Health Conditions, Decision-Making, Mixed-Methods Study, Netherlands

## Abstract

**Background:**

The Netherlands legalised euthanasia and assisted suicide (EAS) in 2002, permitting requests from both physical and mental health conditions under strict conditions. However, physicians are not obliged to comply. General practitioners (GPs), who receive most EAS requests, play a central role in the process of evaluating and granting EAS requests from both patient groups. Although EAS for individuals with a physical health condition is common in the Netherlands, EAS for individuals with a mental health condition remains relatively rare and controversial, despite a growing number of requests. This study explores how Dutch general practitioners’ willingness to grant and perform EAS requests differs between physical and mental health conditions and compares the underlying decision-making processes.

**Methods:**

A concurrent mixed-methods design was employed, combining a quantitative survey and qualitative interviews. The survey was completed by 103 GPs and included sociodemographic and attitudinal questions, experience with EAS, and six randomised case examples varying by physical and mental health condition (cancer, depression) and method (euthanasia or assisted suicide) to examine willingness to grant EAS requests. Semi-structured interviews with 13 GPs explored their reasoning and experiences in more depth.

**Results:**

GPs were significantly less likely to grant EAS requests from individuals with a mental health condition compared to those with a physical health condition (OR = 0.02, 95% CI [0.009–0.04]). Religious GPs were less likely to grant requests (OR = 0.31, 95% CI [0.11–0.85]), and euthanasia was favoured over assisted suicide (OR = 2.3, 95% CI [1.31–4.03]). Diagnosis type and prior experience receiving requests from individuals with a mental health condition showed no significant effect. Willingness to perform EAS was higher for physical health conditions (95.1%) than for mental health conditions (45.6%). Prior experience performing EAS in individuals with a mental health condition was associated with a lower likelihood of restricting willingness to physical health conditions alone (OR = 0.15, 95% CI [0.02–0.73]). Interviews highlighted the greater complexity of EAS in the context of mental health, citing challenges in assessing due care criteria, empathising with requests, ethical dilemmas, extended processes, and lack of confidence. For requests from individuals with a mental health condition, GPs reported requiring additional input from mental health specialists and more often referred these cases to the Expertise Center Euthanasia (ECE).

**Conclusions:**

Dutch GPs are less willing to grant and perform EAS requests from individuals with a mental health condition compared to those with a physical health condition. This may reflect difficulties in assessing due care criteria, clinical uncertainty, challenges in empathising, prolonged processes, and ethical complexity. These findings highlight the need for better implementation of existing guidelines in GP practice, alongside targeted training and stronger support for GPs—including timely access to psychiatric expertise and SCEN consultations, and close collaboration with the Expertise Center Euthanasia (ECE).

**Supplementary Information:**

The online version contains supplementary material available at 10.1186/s12910-025-01333-y.

## Background

The Netherlands was the first country to legalise euthanasia and physician-assisted suicide (EAS) performed by Dutch physicians under the 2002 Termination of Life on Request and Assisted Suicide Act [1]. Euthanasia refers to a physician administering a lethal drug, while assisted suicide involves the physician providing but not administering the drug [2]. Although EAS formally remains a criminal offence under Dutch law, physicians are exempt from prosecution if they act in accordance with the law and meet six due care criteria: (1) the request must be voluntary and well- considered, (2) the patient must be experiencing unbearable suffering with no prospect of improvement, (3) the patient must be fully informed about their situation and prognosis, (4) no reasonable alternative may exist to relieve the suffering, (5) an independent physician must be consulted, and (6) the procedure must be carried out with due medical care and attention [3].

In the Netherlands, physicians must hand in a report to the Regional Euthanasia Review Committees (RTE), which assess whether the physician complied with the statutory due care criteria [4]. In their assessments, the committees make use of the Euthanasia Code, which provides detailed guidance on how the criteria should be interpreted in practice. The Code emphasises, among other requirements, that an independent physician must always be consulted [5]. The Royal Dutch Medical Association (KNMG) has further developed the KNMG Guideline on End-of-Life Decisions, which offers physicians a framework on euthanasia, assisted suicide, and palliative care [6]. In practice, the independent consultation role is often fulfilled by a SCEN physician (Support and Consultation on Euthanasia in the Netherlands). SCEN physicians are specially trained GPs or medical specialists established under the KNMG framework to provide independent consultation and guidance in euthanasia cases [7]. In cases involving mental suffering, the Euthanasia Code 2022 specifies that psychiatric expertise is mandatory, meaning that physicians must consult an independent psychiatrist. Beyond the guidelines and legal framework, the Netherlands also has a specialised institution: the Expertise Center Euthanasia (ECE), formerly known as the End-of-Life Clinic, established in 2012 [8]. The Center provides advice and support to physicians who are handling complex requests. If a physician chooses not to participate in a patient’s request, they can refer the patient to the Center, where trained physicians with expertise in handling EAS requests reassess the case and, if all due care criteria are met, may carry out the procedure.

Thus, individuals with both physical and mental health conditions are legally eligible to have their request for EAS granted. However, EAS in the context of a mental health condition remains rare and controversial. Only a few countries besides the Netherlands, such as Belgium, Luxembourg, Switzerland (for assisted suicide only), and more recently Spain, allow it [9, 10]. In the Netherlands, the number of cases involving mental health conditions rose from 2 in 2008 to 219 in 2024, yet they still accounted for only 2.2% of all reported EAS cases in 2024 [4, 11]. Data from the ECE show that only 10% of requests related to mental health conditions received by the Center are granted, with most declined due to failure to meet the due care criteria, particularly regarding treatment exhaustion and unbearable suffering [12].

Dutch physicians have long reported that EAS requests from individuals with a mental health condition are especially complex and difficult to assess. This is partly due to the greater challenge of applying the due care criteria in the context of mental health conditions, and partly because of limited clinical experience with such requests [13, 14]. Importantly, Dutch physicians are never obligated to grant or perform EAS and may decline for personal or professional reasons [2]. These reasons may influence how requests from individuals with a physical health condition versus those with a mental health condition are handled. Physicians generally find it easier to assess unbearable suffering, prognosis, and decision-making capacity in physical health conditions due to visible physical symptoms and more predictable disease courses. In contrast, suffering related to mental health conditions is often existential, may fluctuate over time, and is frequently accompanied by suicidal ideation or cognitive distortions. These features complicate the evaluation of whether suffering is truly unbearable and without hope of improvement. The ongoing availability of psychiatric treatments further raises doubts about whether such suffering is truly irremediable [15–18]. Beyond clinical challenges, ethical concerns, family involvement, patient attitudes, legal criteria, the physician–patient relationship, and external pressures have all been found to influence EAS decision-making. However, little is known about how these factors differ between cases involving physical and mental health conditions [17, 19–22]. Previous research has shown that physicians are generally more accepting of EAS in the context of physical health conditions than in cases involving mental health conditions [14]. Physician characteristics such as gender, religion, and medical specialty have been identified as important factors. In general, Christian and female physicians, as well as medical specialists, are less likely to find EAS conceivable compared to non-religious, male, and general practitioner (GP). Similar patterns are observed for EAS in the context of mental health conditions, where religious physicians, women, specialists, and psychiatrists in particular report lower conceivability [16, 18].

Among all medical specialties, general practitioners (GPs) are the most open to such cases, with 47% indicating a willingness to perform EAS for individuals with a mental health condition [18]. GPs receive the majority of EAS requests in the Netherlands and, unlike most specialists, often handle both physical and mental health conditions [4, 17]. Their central role makes it especially relevant to examine how they approach different types of requests. For example, Pronk et al. found that 86 out of 101 GPs considered EAS conceivable for individuals with a physical health condition, compared to 51 out of 104 for individuals with a mental health condition. GPs cited values such as compassion, fairness, and respect for autonomy in support of granting requests in cases of mental health conditions, but also expressed hesitation due to perceived medical boundaries, lack of experience, and difficulty applying the due care criteria [14]. However, these data were collected in 2018–2019 and may no longer reflect current views, particularly given that the number of EAS cases related to mental health conditions has doubled in recent years [4].

The incidence of EAS in the context of mental health conditions continues to grow in the Netherlands, fuelling public debate and policy discussions [4]. As global interest in EAS rises, Dutch experiences are becoming increasingly relevant [24]. While previous studies have explored physician attitudes and factors influencing decision-making, most have focused on general conceivability rather than actual willingness to grant or perform requests. Moreover, although interview-based studies with Dutch physicians, particularly psychiatrists have shown that EAS related to mental health conditions is generally perceived as more difficult to evaluate, little is known about how GPs approach and evaluate requests from individuals with a physical versus a mental health condition, and how their decision-making differs between the two. This study addresses that gap by examining current attitudes among Dutch GPs, comparing their willingness to grant and perform EAS across physical and mental health conditions. It also investigates how factors such as the type of mental health condition, the form of EAS, physician experience, and sociodemographic characteristics shape their decisions, and explores differences in the underlying decision-making processes through qualitative analysis.

## Method

### Study design

A concurrent embedded mixed-methods design was employed to combine the strengths of quantitative and qualitative approaches [24, 25]. The quantitative component consisted of a survey designed to gather numerical data and identify broad patterns. It collected information on sociodemographic characteristics including age, religion, years of practice, practice area, gender, and ethnicity, experience with EAS, and attitudes towards euthanasia and assisted suicide (e.g., confidence in guidelines, views on eligibility for individuals with a mental or physical health condition, and preferences for euthanasia versus assisted suicide). The survey also included case examples to examine how factors such as the diagnosis of a mental health condition and the EAS method influenced GPs willingness to grant or deny requests. Survey data were collected in February–March 2025. The qualitative component consisted of 13 online semi-structured interviews with Dutch GPs, conducted in March–April 2025, while the survey was still ongoing. The interviews were embedded within the overall design to provide complementary depth, focusing on the decision-making process, reasoning, and factors that influence GPs’ attitudes towards EAS in both physical and mental health conditions, as well as their willingness to grant requests in both patient groups aspects that could not be fully captured through the survey alone. The interviews therefore provided deeper understanding of the quantitative findings.

### Study population and sampling

The study population consisted of 103 general practitioners (GPs) who fully completed the questionnaire. In total, 609 GP practices were contacted and 111 GPs responded. Eight responses were excluded because respondents left whole sections of the questionnaire unanswered, resulting in a final sample of 103 GPs. Since GP practices rather than individual GPs were contacted directly, an exact response rate cannot be calculated. However, the sample represents approximately 17% of the approached practices. Interview participants were purposively recruited through survey responses, personal and professional contacts, and snowball sampling, with the goal of capturing variation in gender, region, and experience. Recruitment continued until no further relevant information emerged in the final interviews. The final qualitative sample comprised 13 GPs (11 female, 2 male), including two SCEN physicians, all actively practising in various regions of the Netherlands. Four interviewees contacted the researcher via the survey to participate and therefore completed both the survey and the interview. The remaining interview participants were recruited through direct contact or snowball sampling, and for these cases it is uncertain whether they also completed the survey.

### Data collection

For the quantitative component, the online survey provided in Additional file 1 was designed using LimeSurvey and distributed via email to Dutch GP practices in February and March 2025. It included closed questions assessing experience, attitudes towards euthanasia and assisted suicide (EAS) for individuals with physical and mental health conditions, willingness to perform EAS, and sociodemographic characteristics. Survey questions were either directly taken from the fourth evaluation of the Dutch Euthanasia Act or were partly self-developed, based on questions from the evaluation but adapted to fit the specific aims of this study [17]. An overview of the variables targeted by the survey questions is presented in Table 1, and a summary of the attitude-related items is provided in Table 5. The cut-off values used for age and years of practice in this study are based on categorizations applied in previous research on physicians’ attitudes toward euthanasia [18, 26].

**Table 1:** Overview of the variables included in the regression analyses, along with their coding schemes

Variable	Description	Variable coding	Regression models used
*Dependent*			
Combined willingness	Willingness of GPs to perform EAS themselves	0 = Willing for both physical and mental request 1 = Only willing for physical requests	Binary logistic (Willingness to perform)
Grant requests	Whether the GP would grant the requests for the case studies	1 = yes 0 = No	Multilevel (case examples)
*Independent*			
Age	Age group of the respondents	Categorical: < 40 (reference) 40–54 and > 50	Binary logistic & Multilevel
Religion	Religious affiliation	1 = Religious 0 = Non-religious	Binary logistic & Multilevel
Years of practice	Years of practice as a GP	1 = > 10 0 = 2–10	Binary logistic & Multilevel
Practice area	Area of practice of GP	1 = Rural 0 = Urban	Binary logistic & Multilevel
Experience receiving EAS requests for patients with mental health conditions only	Experience receiving EAS requests from patients who suffer mentally	1 = Yes 0 = No	Binary logistic
Experience performing EAS for patients with mental health conditions	Experience performing EAS for people suffering mentally	1 = Yes 0 = No	Binary logistic (Only willingness to perform model)
Request type	Indicates whether the condition is physical or mental	1 = mental, 0 = physical	Multilevel
EAS type	Type of EAS request	1 = Euthanasia, 0 = Assisted suicide	Multilevel
Condition type	Type of mental health condition	Categorical: Autism (reference) depression, schizophrenia and PTSD	Binary logistic (Model 4 & 5 case examples)

Gender was excluded from analysis due to a high rate of missing responses. Ethnicity, experience with EAS requests in physical health conditions, and performing EAS in physical health conditions were excluded from analysis due to insufficient variability in the data. The variable for performing EAS in cases of mental health conditions was excluded from the analysis of the mental health case examples, as the very low number of GPs with such experience led to instability in estimates. All case examples on mental health conditions themselves were included in the analysis. Practice area was assessed as a self-reported variable, which may not fully align with official classifications regarding urban and rural in the Netherlands

The survey also employed a case example approach to explore GPs’ willingness to grant requests. Twelve case examples were developed, with each GP completing six randomly selected cases (Table 2) that varied by request type (physical or mental health condition), EAS method (euthanasia or assisted suicide), and mental health condition diagnosis (autism, depression, schizophrenia, PTSD). The cases were developed based on examples from the fourth evaluations of the Dutch Euthanasia Act and were further inspired by the work of Kouwenhoven et al. [26].

**Table 2:** Illustrative set of randomly assigned case examples completed by GPs

Case examples
1. Mr. Van de Berg suffers from ALS. He has lost nearly all motor functions, including the ability to walk, speak, and swallow independently. Despite receiving supportive care, he is completely dependent on others for all daily activities. His condition will inevitably lead to respiratory failure. He finds his current and future situation unbearable and submits a request for euthanasia
2. Ms. De Jong has metastatic breast cancer. She has undergone several treatments, but her disease is no longer curable. She experiences severe, hard-to-manage pain and feels she is losing control over her life, a sense of control she valued deeply during her working life. She states she cannot go on like this and requests euthanasia
3. Ms. De Jong has metastatic breast cancer. After various treatments, her illness has become incurable. She suffers from intense pain and feels a loss of control over her life, which is deeply distressing for her. She says she can no longer cope and asks her GP for a life-ending drug that she can take herself
4. Mr. Jansen has lived with schizophrenia for many years. Despite consistent treatment with medication and therapy, he continues to experience severe hallucinations and delusions that significantly impair his quality of life. These symptoms cause him immense suffering, and he sees no hope for improvement. After careful consideration, he requests euthanasia from his GP
5. Ms. Langezaal is physically healthy but suffers from severe, long-term depression. Psychiatric treatments have failed to relieve her symptoms. She frequently tells her doctors she wants to die and has previously attempted suicide, unsuccessfully. She asks her GP for euthanasia to end her suffering
6. Ms. Smit suffers from severe ASD, which has led to lifelong unbearable suffering. She endures continuous sensory overload, isolation, and unrelieved emotional distress. Years of treatment have failed to help her. She is unable to engage in everyday life or social contact and finds the overstimulation unbearable. She asks for a life-ending drug she can take herself to end her suffering

To further explore decision-making processes, 13 semi-structured interviews were conducted using an interview guide. The interview guide (Additional file 2) was developed on the basis of a literature search using Google Scholar and PubMed, including previous studies on EAS and the decision-making processes of Dutch physicians. Interviews were held online via Zoom, except for two that were conducted by telephone due to scheduling constraints. For all interviews the interviewer (first author) recorded the audio via the Dictaphone app. The interviews explored GPs’ experiences, external influences, and perceived barriers in evaluating both physical and mental health EAS requests. Each session lasted approximately 33 min (range 24–43 min) and provided qualitative insights into the factors shaping GPs decisions across request types. All interviews were conducted in Dutch by the first author, who has prior experience with interviewing on sensitive topics and followed multiple courses in qualitative research. As a Dutch citizen, the author approached the subject from an academic and inquisitive standpoint. Interview questions were formulated and asked in a neutral manner. Reflexive attention was maintained throughout the interviews, with the interviewer explicitly considering her own neutrality and ensuring neutrality in both her questioning and her interpretation of the information. In this manuscript, the original terms used in the survey and interview quotes (“psychiatric” and “somatic”) have been retained verbatim; throughout the text, “psychiatric” refers to mental health conditions and “somatic” to physical health conditions.

### Data analysis

Survey data were analysed using IBM SPSS Statistics 28 and R version 4.4.1. Descriptive statistics (frequencies and percentages) were used to assess demographics, attitudes, and experiences. Case example responses were analysed using multilevel and binary logistic regression in R, with the dependent variable being whether the GP indicated they would grant the requests from the case examples (yes/no). Five models were constructed. Since each GP assessed six randomly selected case examples, multiple responses were nested within individual participants, introducing potential clustering. Responses from the same GP were likely to be more similar than those from others. The intraclass correlation coefficient (ICC = 0.162) confirmed that 16.2% of the variance in willingness was attributable to between-GP differences, exceeding the commonly applied 10% threshold [27]. Therefore, multilevel modelling was applied in Models 1–3.

Across all models, the dependent variable was whether the GP indicated they would grant the request in each case example (yes/no). Model 1 included demographic characteristics (age group, years in practice, practice area, and religion). Model 2 added request type (physical or mental health condition), and Model 3 further included EAS method (euthanasia or assisted suicide). For Model 3, the bobyqa optimiser (15,000 iterations) was applied to address convergence issues. Models 4 and 5 focused specifically on mental health condition cases, examining condition type and experience with receiving mental health condition requests. Due to convergence issues and unstable estimates for condition type and experience receiving mental health condition requests, binary logistic regression was used for Models 4 and 5. Odds ratios (ORs) and confidence intervals (CIs) for other predictors remained stable across models, so this adjustment did not affect interpretation. Some variables were excluded due to missing data (gender), low variability (ethnicity, physical health condition EAS experience), or estimation instability (experience performing requests related to mental health conditions). Religion and years of practice were recoded into binary variables due to low category frequencies. All analyses were performed in R using the packages haven (2.5.4), readr (2.1.5), dplyr (1.1.4), stats (4.4.1), tidyr (1.3.1), ggplot2 (3.5.2), nnet (7.3–20), lme4 (1.1–37), broom (1.0.8), boot (1.3–30), and performance (0.13.0).

Additionally, a separate binary logistic regression analysed willingness to perform EAS requests in R. The original three-category outcome (willing to perform both EAS requests from physical and mental health conditions/willing to perform physical health condition only/unwilling for both) was reduced to a binary variable by excluding the small ‘unwilling’ group. This resulted in a comparison between those willing to perform physical health condition EAS only and those willing to perform both physical and mental health condition EAS. The same variable exclusions applied as in the previous models, except that experience performing EAS for individuals with a mental health condition was retained in this analysis, as convergence issues did not arise for this variable.

For the qualitative part, interviews were transcribed verbatim and analysed in MAXQDA 24 using qualitative content analysis, applying a combination of directed and conventional approaches [28]. This hybrid coding strategy ensures a structured yet flexible approach to analysis, grounded in theory but open to new insights [29]. The coding framework was developed in several phases. First, deductive codes were generated from the interview guide, which itself had been informed by a review of the literature on EAS decision-making. Second, the transcripts were read repeatedly to ensure familiarisation, during which additional inductive codes were created to capture recurring information that emerged directly from the data. Third, main codes and subcodes were developed, and a distinction was made between physical and mental health conditions to allow systematic comparison of decision-making processes. Fourth, all interviews were coded using this combined deductive–inductive framework (see Additional File 3 for the coding scheme). Fifth, summaries of each interview were produced with the assistance of MAXQDA’s AI summary tool in English, which supported data familiarisation but did not replace the manual coding process. Finally, the first author summarised and interpreted the coded segments to highlight patterns across interviews and identify key factors influencing decision-making.

## Results

### Quantitative results

#### Survey respondents’ characteristics

Table 3 presents the demographic characteristics of the GPs who completed the survey. It is important to note that not all participants provided complete demographic data. As a result, the number of respondents varies slightly across variables, and totals differ between questions. Of the respondents who reported their gender, 40 were female and 25 male. For age, 16 were under 40 years, 58 were between 40 and 54 years, and 29 were over 55 years. Regarding religion, 30 identified as religious and 72 as non-religious. In terms of years of practice, 14 had worked as a GP for 2–10 years, and 87 for more than 10 years. For practice area, 67 worked in rural areas and 34 in urban practices. Finally, almost all participants were of Dutch ethnicity (101), with only 2 reporting a non-Dutch background.

**Table 3:** Demographic and background characteristics of survey respondents

*Background characteristics (n* = *103)*	N	Valid %
Gender		
Female	40	61.5
Male	25	38.5
Age		
< 40	16	15.5
40–54	58	56.3
> 55	29	28.2
Religion		
Religious	30	29.1
Non-religious	72	69.9
Years of practice		
2–10	14	13.9
> 10	87	86.1
Practice area		
Urban	34	33.7
Rural	67	66.3
Ethnicity		
Dutch	101	98.1
Non-Dutch	2	1.9

Percentages are calculated based on valid responses for each characteristic. Missing values were excluded from the denominators, leading to varying totals across variables

#### GPs’ experience and attitudes regarding EAS

Most GPs who participated in the survey had experience with receiving EAS requests based on a physical health condition only (101). When asked about performing EAS for individuals with a physical health condition, 97 GPs reported that they had performed it before, while 5 had not. For requests related to mental health conditions, 72 GPs had previously received a request based solely on a mental health condition, and 11 reported having experience performing EAS for such individuals (Table 4).

**Table 4:** Respondents’ experience with receiving EAS requests and performing EAS

*Experience*	N	Valid %
Received physical-only EAS request		
Yes	101	100
No	-	-
Performed EAS – physical basis only		
Yes	97	95%
No	5	5%
Received mental condition-only EAS request		
Yes	72	70%
No	31	30%
Performed EAS – mental condition basis only		
Yes	11	10.7%
No	92	89.3%

“Only” refers to the fact that the EAS request stemmed solely from either a physical health condition or a mental health condition, without influence from a combination of conditions or other contributing factors. Percentages are based on valid responses only; missing values were excluded from the denominator

GPs’ attitudes toward EAS were measured (Table 5) for both general and specific patient types (physical and mental health conditions). Most GPs expressed the strongest agreement with feeling less confident when assessing EAS requests from individuals with a mental health condition, with 70 strongly agreeing. Agreement that individuals should be eligible for EAS was highest for physical health conditions (39 strongly agreed, 50 agreed), whereas responses regarding mental health conditions showed more variation (17 strongly agreed, 52 agreed). Still, a majority of GPs agreed that individuals with a mental health condition should have the same opportunity to request EAS as those with a physical health condition, with 37 agreeing and 16 strongly agreeing. The findings from the statements suggesting it is impossible to assess unbearable and irremediable suffering, or to determine whether the request is well-considered in individuals with a mental health condition, show that most GPs either held a neutral point of view or disagreed with these statements, with 35 neutral and 43 disagreeing on the statement regarding suffering, and 32 neutral and 47 disagreeing on the statement regarding the request being well-considered. Confidence in current guidelines was low, with 35 GPs disagreeing that they provide sufficient clarity for EAS in the context of mental health–related suffering and 6 strongly disagreeing. Most GPs also did not favour assisted suicide over euthanasia for either patient group. Instead, most showed a tendency to favour euthanasia over assisted suicide for both patient groups, as the majority did not indicate a greater willingness to provide assisted suicide.

**Table 5:** GPs attitudes toward EAS requests from patients suffering from physical and mental health conditions

*Attitudes Dutch GPs towards EAS*	Strongly disagree (1)	Disagree (2)	Neutral (3)	Agree (4)	Strongly agree (5)	Mean score	Total N
I feel less confident when assessing EAS requests from psychiatric patients compared to patients with somatic conditions	3	2	5	21	70	4.51	101
I believe that patients with somatic conditions should be eligible for EAS if they request it	3	2	8	50	39	4.18	102
I believe that patients with psychiatric conditions should be eligible for EAS if they request it	3	7	22	52	17	3.72	101
Everyone has the right to decide about their own life and death	4	17	21	39	20	3.53	101
Psychiatric patients should have the same opportunity to request EAS as patients with somatic conditions	5	14	28	37	16	3.45	100
It is impossible to determine whether a psychiatric patient’s wish to die stems from psychopathology	1	40	39	17	4	2.83	101
The current guidelines provide sufficient clarity to help me assess EAS requests from psychiatric patients	6	35	29	24	1	2.78	95
It is impossible to assess whether a psychiatric patient is suffering unbearably and without prospect	6	43	35	10	5	2.65	99
It is impossible to determine whether a psychiatric patient’s wish to die is well-considered	8	47	32	11	3	2.54	101
I would be more willing to provide assisted suicide than euthanasia to psychiatric patients	26	33	11	30	2	2.50	102
I would be more willing to provide assisted suicide than euthanasia to somatic patients	31	33	17	17	4	2.31	101

The mean scores reflect the average level of agreement with each statement on a 5-point Likert scale. Higher scores indicate stronger agreement. The term “psychiatric” is retained to reflect the original wording of the survey question. In the context of this study, “psychiatric” refers to mental health conditions, the clarification: “and ‘somatic’ refers to physical conditions.

#### GPs willingness to grant EAS requests and influencing factors

Table 6 presents the results from the multilevel regression models (Models 1–3) and binary logistic regression models (Models 4–5), which were based on GPs responses to the randomly assigned case examples and GPs previous experience with receiving requests from individuals with a mental health condition. Model 1 included only sociodemographic variables, with religion emerging as the sole significant predictor: religious GPs were less likely to grant requests than non-religious GPs. Age, years of practice, and practice area showed no significant effects. Model 2 added request type and showed that requests based on mental health conditions were significantly less likely to be granted than those based on physical health conditions, while religion remained significant. Model 3, the most comprehensive model, included all prior variables and added EAS method. In Model 3, three variables were significantly associated with the likelihood of granting requests: (1) requests from individuals with a mental health condition were substantially less likely to be approved compared to requests from those with a physical health condition (OR = 0.02, 95% CI [0.009–0.04]); (2) euthanasia requests were more likely to be granted than assisted suicide requests (OR = 2.3, 95% CI [1.31–4.03]); and (3) religious GPs were less likely to grant requests than non-religious GPs (OR = 0.31, 95% CI [0.11–0.85]). Models 4 and 5 focused specifically on requests related to mental health conditions, examining whether the type of condition (Model 4) or prior experience with such requests (Model 5) influenced willingness to grant. Neither model showed statistically significant effects.

**Table 6:** Overview of the results from the case examples analyses using multilevel and binary logistic regression

	Model 1 Demographics (Multilevel)		Model 2 physical vs. Mental health (Multilevel)		Model 3 Euthanasia vs. assisted suicide (Multilevel)		Model 4 Condition type (Binary)		Model 5: Mental suffering experience (Binary)	
	OR	CI (95%)	OR	CI (95%)	OR	CI (95%)	OR	CI	OR	CI (95%)
Intercept	2.7	[1.27–5.83]	32.1	[7.75–132.8]	23	[5.23–101.3]	0.88	[0.32—2.30]	1.08	[0.38–3.06]
Age										
< 40	1	1	1	1	1	1	1	1	1	1
40–55	0.95	[0.35–2.57]	0.74	[0.13–4.10]	0.84	[0.14–5.03]	0.40	[0.14–1.15]	0.40	[0.14–1.14]
> 55	1.1	[0.41–3.10]	1.1	[0.20–6.47]	1.28	[0.21–7.90]	0.84	[0.30–2.35]	0.87	[0.31–2.44]
Religion										
Non-Religious	1	1	1	1	1	1	1	1	1	1
Religious	0.46*	[0.26–0.80]	0.32*	[0.12–0.84]	0.31*	[0.11–0.85]	0.62	[0.32–1.14]	0.59	[0.39–1.10]
Years of practice										
2–10	1	1	1	1	1	1	1	1	1	1
> 10	0.77	[0.28–2.17]	0.78	[0.13–4.58]	0.71	[0.11–4.57]	1.09	[0.37–3.24]	1.15	[0.39–3.44]
Practice area										
Rural	1	1	1	1	1	1	1	1	1	1
Urban	0.87	[0.51–1.50]	0.77	[0.30–1.95]	0.77	[0.29–2.05]	0.88	[0.47–1.61]	0.90	[0.48–1.66]
Request type										
physical			1	1	1	1	1	1	1	1
Mental			0.02*	[0.01–0.04]	0.02*	[0.009–0.04]	-	-	-	-
EAS type										
Assisted suicide					1	1	1	1	1	1
Euthanasia					2.3*	[1.31–4.03]	0.62	[0.36–1.09]	0.63	[0.36–1.10]
Condition type										
Autism							1	1	1	1
Depression							0.82	[0.34–1.92]	0.82	[0.34–1.92]
PTSD							1.75	[0.83–3.78]	1.74	[0.82–3.76]
Schizophrenia							1.19	[0.55–2.56]	1.15	[0.54–2.49]
Received mental condition-only EAS request										
Yes									1	1
No									0.69	[0.37–1.28]

Multilevel logistic regressions used for model 1–3 and binary logistic regressions for model 4 & 5

^*^significant results 1 = reference category

#### Willingness to perform EAS requests: mental versus physical health conditions

**Fig. 1:**
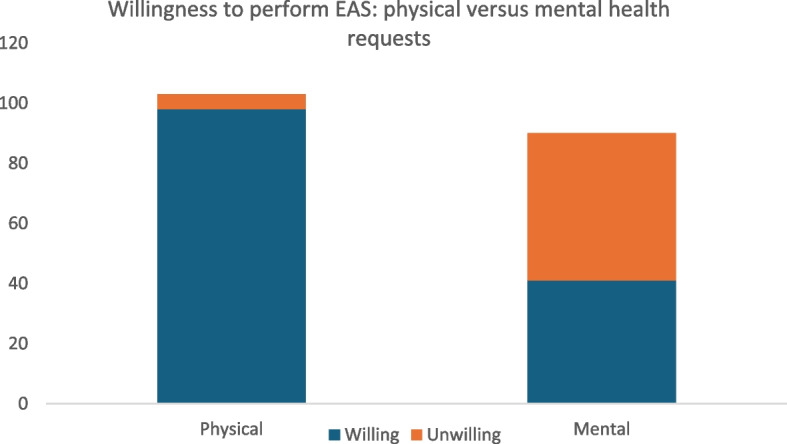
Willingness to perform EAS: physical versus mental health conditions. Note: Values represent the number of GPs who indicated whether they were willing or unwilling to perform EAS. N = 103 for physical health conditions and 90 for mental health conditions

When asked directly about their willingness to perform EAS via the survey, 98 GPs indicated willingness for individuals with a physical health condition, while 5 were unwilling (Fig. 1). For individuals with a mental health condition, 41 GPs were willing, whereas 49 were unwilling.

Results of the binary logistic regressions comparing willingness to perform only physical health condition EAS requests with willingness to perform both physical and mental health condition requests are presented in Table 7. The only significant predictor was prior experience performing EAS requests for individuals with a mental health condition (OR = 0.15, 95% CI [0.02–0.73]), indicating that GPs with such experience were more likely to also be willing to perform requests in these cases. Other variables, including age, religion, years of practice, practice area, and experience receiving requests related to mental health conditions, were not statistically significant. Variables on physical health condition EAS experience and ethnicity were excluded due to lack of variability, and gender was excluded due to insufficient responses. However, results from the subgroup that did report gender were consistent with the overall model, suggesting no gender effects.

**Table 7:** Results of the binary logistic regression analysis on GPs willingness to perform

	Binary Logistic Regression Model	
	OR	CI (95%)
Intercept		
	0.53	[0.08, 3.42]
Age		
< 40	1	1
40–55	1.57	[0.24, 10.42]
> 55	1.54	[0.23, 10.52]
Religion		
Non-religious	1	1
Religious	1.17	[0.41, 3.41]
Years of practice		
> 10	1	1
2–10	0.56	[0.07, 4.30]
Practice area		
Rural	1	1
Urban	0.67	[0.23, 1.91]
Received mental health condition-only EAS request		
No	1	1
Yes	2.47	[0.85, 7.53]
Performed EAS – mental health condition basis only		
No	1	1
Yes	0.15*	[0.02, 0.73]

### Qualitative results

#### The process of decision-making: Dutch GPs and euthanasia requests

A total of 13 Dutch physicians, all qualified as GPs and with multiple years of experience in general practice, participated in the interviews. Eleven were still working as GPs at the time of the interview, while two were currently working as SCEN physicians (Table 8). All 13 GPs interviewed stated that their experience involved euthanasia requests, whether related to physical or mental health conditions, and none had experience with assisted suicide. Therefore, their decision-making process is relevant solely to euthanasia and not assisted suicide. However, when discussing assisted suicide as an option many GPs expressed being more hesitant or reluctant towards this method. As stated by two GPs: “*I must say, because I did not see it during my training and have never experienced it, if a patient were to ask me, ‘I would really like to do it myself,’ I would certainly honour that request, but I would personally feel a bit more tense.*” (P3) and “*So far, I have not had any experience with assisted suicide, but I have with euthanasia. The reason for this is that a colleague in my group had an unpleasant experience with assisted suicide about ten years ago, because at that time the dosage of the drink was much lower than it is today. I am also someone who likes to have control, and you have much more control with euthanasia and less with assisted suicide.”* (P6).

**Table 8:** Demographic and professional characteristics of the interview participants

Participant	Gender	Practice area	GP in current practice	Experience euthanasia
1	Female	Urban	Trained as GP, currently practising as SCEN physician	Received and performed euthanasia for patients suffering from physical and mental health conditions
2	Female	Rural	Yes	Received requests from patients suffering from physical and mental health conditions. Has never performed euthanasia
3	Female	Rural	Yes	Received requests from both patient groups, only performed requests from patients with physical conditions
4	Male	Urban	Yes	Received requests from both patient groups, only performed requests for patients with physical conditions
5	Female	Rural	Yes	Received requests from both patient groups, only performed requests from patients with physical conditions
6	Female	Urban	Yes	Received and performed euthanasia for patients suffering from physical and mental health conditions
7	Female	Urban	Yes	Received requests from both patient groups, only performed requests from patients with physical conditions
8	Female	Urban	Yes with an accredited specialization in elderly care	Received requests from both patient groups, only performed requests from patients with physical conditions
9	Female	Urban	Yes, also practising as a locum GP	Received and performed euthanasia for patients suffering from physical and mental health conditions
10	Female	Urban	Yes	Received requests from both patient groups, only performed requests from patients with physical conditions
11	Female	Urban	Yes	Received and performed euthanasia for patients suffering from physical and mental health conditions
12	Female	Urban	Trained as GP, currently practising as SCEN physician	Received requests from both patient groups, only performed requests from patients with physical conditions
13	Male	Urban	Yes	Received requests from both patient groups, only performed requests from patients with physical conditions

Trained as GP refers to the fact that the participant completed formal education and training required to become a GP

All GPs were open to discussing euthanasia with their patients, and twelve expressed general willingness to perform it. Their decision-making was nuanced and influenced by multiple elements. Most reported that euthanasia consultations are often initiated as early conversations regarding advance care planning, in which patients explore their options and express interest in considering it as a future possibility: “*So that first conversation is more of a hedging than really a direct question of ‘I want it to happen.*’” (P4). These early conversations aim to inform and clarify patient wishes rather than initiate the formal process. When individuals with serious health conditions (i.e. severe, life-limiting illnesses that could potentially qualify for euthanasia, rather than minor ailments such as a cold) make an actual request, GPs begin deliberating on the best course of action.

#### Importance of the doctor–patient relationship

A recurring theme was the importance of a long-term patient relationship. Many GPs stressed that decisions unfold over time, not in a single consultation: *“That is not a decision made over one night, but during the course of someone’s illness.*” (P7). Initially, GPs focus on supporting and informing the patient, often explaining end-of-life care options such as palliative sedation. If a patient remains certain about euthanasia, GPs assess the legal criteria, including suffering and whether the request is well-considered: “*I want to be clear about what unbearable suffering consists of, so I really let someone articulate themselves.*” (P6). In addition to supporting, informing, and evaluating whether the request meets the criteria set by law, the interviews made it clear that GPs navigate the moral implications of ending someone’s life, even when a patient is suffering. One GP expressed that they could not perform euthanasia at all, regardless of the case, due to personal beliefs: “*I couldn’t even kill a mosquito when I was younger, so I’m certainly not going to kill a person. I will do everything to alleviate suffering and help during the final stage, but I won’t do that.*” (P2). When discussing different cases and GPs personal boundaries, many GPs expressed that if they are unable to perform euthanasia themselves, they are relieved that the Expertise Center Euthanasia (ECE) exists, allowing them to refer their patients there.

#### Ethical considerations in GPs decision-making

Even for those who do perform euthanasia, GPs reported engaging in significant moral reflection before making a final decision. As several GPs explained: “*You must be able to justify it to yourself*.” (P1) and “*Does it feel right to do this for this patient? Because I have to live with the knowledge that I will do this and have done it at some point. And I have to live with the absolute certainty*.” (P13). Many cited the relief of unbearable suffering and providing a dignified death as central to their moral justification: “*If someone suffers unbearably, irremediable, and I cannot relieve it in any other way, then I see euthanasia as the last therapy*.” (P6). Autonomy was another prominent theme. GPs stated that they deeply respect patients right to decide how and when to die: “*I believe that everyone has the free choice, and my conviction should not be an obstacle to that. Everyone has the right to freely choose how they live and end their life.*” (P2). As they value patient autonomy, GPs also assess throughout the process whether the request is well-considered and voluntary, and not influenced by family or friends: “*And then I always find it important for myself that I speak to the patient alone – because often there are family members present – to make sure that it is indeed the person’s own wish and not something imposed by the surroundings.*” (P6). Trust-building and empathy were also cited as essential. GPs use consultations not only to assess criteria but to determine whether they can empathise with the patient’s wish. This was articulated clearly by multiple GPs: “*But I personally feel that I must also be able to empathise with it in order to do it. It's not nothing to do*.” (P7) and “*And then the request comes very late, and I can't fully empathise with it in just three days.*” (P3).

#### Euthanasia as a complex and demanding process

GPs emphasised that the process behind exploring a euthanasia request is complex and must not be underestimated. One GP detailed the process from start to finish: “*This is something you genuinely have to support wholeheartedly, but it’s a process that I handle very carefully. You have multiple conversations until you decide to bring in a second doctor because you feel ready to proceed with the euthanasia. Then the SCEN physician comes, and they give the green light. And that it is indeed a situation of irremediable, unbearable suffering. Then the protocol actually begins. The protocol that you will follow, the date that is scheduled, the medications that need to be ordered from the pharmacy, the reports that have to be filled out in advance. It’s a whole process leading up to it.*” (P9). Other GPs described the process as “intense” and “energy-consuming.” Beyond conversations and decision-making, the administrative burden was also noted, especially the documentation required by the RTE (Regional Euthanasia Review Committee), with one GP stating: “*I always say that euthanasia is a legal process, not a medical process.*” (P2). Besides practical complexities, GPs reported significant emotional strain. One GP stated: “*I find it hard. The conversations you have are intense and take a long time.” (P6). The emotional weight increases in the final stages. GPs reported sleepless nights, lingering stress, and emotional recovery time: “The week leading up to the euthanasia is difficult for me. In the sense that it occupies my mind a lot and I sleep poorly.*” (P8) and “*You have to take an afternoon off to do it, and then you need some time to recover in the evening. And it still lingers in your mind for a week afterward.*” (P2).

#### Family influence and professional support

While the patient’s wish is central, most GPs said they also consider the views of family members. As one GP put it: “*It mainly concerns the patient, but it does help if the family is also supportive.*” (P6). In cases where family support is absent or conflicting, GPs respond differently. Some are troubled by family disagreement, while others feel it is up to the patient and family to resolve such conflicts themselves. In either case, family disagreement can make the process of evaluating a request more difficult. A few GPs stated that family opinions do not influence their decision at all. To cope with the emotional and procedural burden, GPs often seek support. Many stressed the importance of having a colleague or trainee present, with one GP indicating that performing euthanasia alone would not feel right: “*I never do it alone, there's always a colleague or my buddy with me. And I’ve also joined others on their routes. I’m really not going to do it by myself if it doesn’t feel right or anything, it’s all just terrifying.*” (P10). GPs also consult other professionals, such as palliative care nurses and SCEN physicians, for guidance and emotional reinforcement.

#### Comparing decision-making processes: mental versus physical health conditions

The decision-making process surrounding euthanasia requests differs significantly between physical and mental health conditions, as revealed through interviews with GPs (Table 9). While some GPs expressed openness to evaluating both: “*It doesn't matter to me whether someone has metastatic lung cancer or indeed an incurable depression.*” (P13) others viewed requests from individuals with a mental health condition as outside their professional scope: “*I don't believe that it falls within the GP's role.*” (P4). Opinions varied: some had experience with mental health condition cases, some were open to them, while others excluded this type of request entirely. However, all agreed that requests related to mental health conditions are generally more complex.

**Table 9:** Comparison of the decision-making process of Dutch GPs for mental versus physical health requests for EAS

*Comparison Decision making process of Dutch GPs (n* = *13)*	Physical health conditions	Mental health conditions
Openness to evaluate requests	All GPs (except 1) were open to evaluating euthanasia requests	Opinions varied: some open, others excluded mental health requests entirely
Unbearable suffering	More visible, measurable, and often linked to clear symptoms (e.g., pain, shortness of breath), supported by specialist documentation	Harder to assess; suffering less measurable and often more subjective; difficulties in interpretation and articulation by patients
Irremediability/treatment exhaustion	Often straightforward, supported by clear documentation from specialists confirming no further treatment options	Greater uncertainty: lack of fixed protocols, unpredictable outcomes, doubts about whether all reasonable treatments have been tried
Well-considered and voluntary decisions	Requests generally seen as stable and easier to verify as voluntary and well-considered	Requests often unstable over time; illness itself may impair decision-making; harder to separate illness-driven death wish from genuine suffering
Knowledge and expertise needs	GPs felt more capable and confident	GPs emphasised need for psychiatric expertise or external input
Referral behaviour	Referral to ECE mentioned less frequently	Referral to ECE or need for consultation was more common
Ethical reflection	General moral reflection noted	Additional ethical concerns: societal failures (e.g., loneliness, lack of care), personal boundaries, religious beliefs
Empathy	GPs are often able to empathise with physical suffering	Some struggled to emotionally connect despite recognizing real suffering
Process time	Seen as faster and more straightforward	Perceived as more time-intensive and requiring more consultation
Fear of legal prosecution	Rarely mentioned; low concern	Some indication of fear but rarely mentioned
Guidelines and law clarity	KNMG guidelines widely used and understood	Less familiar with guidelines from NVvP, often not used by GPs
Influence of patient characteristics	Younger patients with families raised some concern	Youth often seen as a barrier; more hesitation reported

#### The complexity of assessing unbearable suffering for mental health conditions

A key challenge in euthanasia for mental health conditions is determining whether the patient’s suffering is genuinely "unbearable" and " irremediable." Throughout the interviews, GPs indicated that it is harder to determine whether the suffering is unbearable for individuals with a mental health condition. One GP expressed difficulties examining this since patients have trouble expressing and describing their suffering: “*And the unbearableness is sometimes very difficult to put into words. Yesterday I visited a psychiatric patient, a simple man who therefore had difficulty expressing the unbearableness.*” (P1). Another GP further indicated that unbearable suffering is easier to examine for individuals with a physical health condition compared to those with mental health conditions: “*It is of course much easier if someone is in a lot of pain, or very short of breath, or does not want to be dependent on care. Those are clear reasons in somatic cases, so you reach a conclusion much sooner than with psychiatry.*”

#### The complexity of assessing irremediable suffering in mental health conditions

GPs also emphasised the difficulty of establishing irremediable suffering in mental health conditions. As one GP expressed: “*In psychiatry, I find the irremediability very difficult.*” (P8), and another GP indicated that irremediability is harder to objectify: “*What I find difficult is that the judgment of whether something is treatable or irremediable is generally much harder to objectify in psychiatry.*” (P13). Furthermore, uncertainties in determining irremediability for mental health conditions were highlighted: “*Psychiatric suffering is difficult in that sense, because compared to most somatic conditions, it’s harder to determine. When can we all be certain that this condition is an irremediable problem that we can no longer do anything about medically?*” (P13). Establishing whether all reasonable, available, and indicated therapeutic options for a patient’s condition have been tried (treatment exhaustion) is in practice often linked with determining irremediability. Treatment exhaustion was described as more straightforward in physical health conditions, as it is usually backed by specialist documentation: “*You just have a letter from an oncologist that says this is the prognosis, and the patient is out of treatment options. And that’s very black and white.*” (P3). In mental health cases, the absence of fixed protocols and the unpredictability of outcomes created more uncertainty. As one GP expressed: “*What I find hard is that the judgment of whether something is treatable or irremediable is sometimes harder to objectify in psychiatry*” (P13).

#### The combination of unbearable suffering and irremediability

Although unbearableness and irremediability are distinct legal criteria, GPs often experience them as closely intertwined. In practice, the two reinforce one another, as irremediability may make suffering more unbearable, and their overlap contributes to the overall difficulty of assessing suffering in mental health conditions. In general, GPs expressed that suffering related to physical health conditions is more clearly defined and straightforward, as it is usually backed by specialist documentation that helps to determine both the unbearableness and irremediability: “*You just have a letter from an oncologist that says this is the prognosis, and the patient is out of treatment options. And that’s very black and white.*” (P3). Another GP stated: “*People also come back from a specialist, like the pulmonologist or cardiologist, and are out of treatment options. Somehow, I feel that’s different from psychiatry. It’s not as objective because you can't measure it in the same way. With tumours, you can measure it.*” (P11). Moreover, GPs expressed greater uncertainty when assessing suffering in mental health conditions, as one questioned: “*How sure can you be that these complaints really can’t improve in any way?*” (P13). While GPs acknowledged this complexity, most agreed that suffering related to mental health conditions can indeed be irremediable and unbearable, though harder to assess.

#### Well-considered and voluntary requests in mental health conditions

GPs emphasised that when handling euthanasia requests from patients with mental health conditions, they make clear that those requests must meet the criteria of being well-considered and voluntary: “*At a certain point he said to me: if I really become demented, then I want euthanasia. And then I said: yes, I will be there for you, provided you can make it clear that you want that, that it is your choice*.” (P11). However, GPs expressed that for mental health conditions this is harder to establish due to requests often being unstable over time: “*Like that one patient with a personality disorder and also depressive symptoms, who turned out to be able to change in that regard after all. So in the end, not very stable.*” (P9). Additionally, the illness itself can cause psychopathology, meaning that symptoms of the mental disorder (such as delusions, compulsions, or severe mood disturbances) directly influence the patient’s thoughts, feelings, and decision-making processes. This makes it more difficult to determine whether the request is truly voluntary and well-considered. As one GP explained: “*Because it’s hard to distinguish between what is the illness and what is not the illness, does a death wish stem from their illness, or from their unbearable suffering?*” (P6). This makes assessment more difficult than in physical health condition cases, where wishes are usually clearer.

#### The challenge of uncertainty and ethics in mental health suffering

Because of the challenges in assessing the suffering and decision-making capacity of patients with mental health conditions, many GPs expressed hesitation due to their limited expertise in psychiatry. Even those willing to proceed emphasised the need for specialist support. When asked about assessing such requests, one GP stated: “*No, I can't do that on my own, so I ultimately need the support of specialists in that area, much more than with somatic conditions.”* (P13). The need for psychiatric input was a common theme: “*In that case, I really need the expertise of a psychiatrist to look into it as well. To determine what comes from the illness and what comes from the wish to die, and what is still treatable in that?”* (P5). In contrast, most GPs felt more confident in evaluating requests related to physical health conditions: “*For somatic patients, I can handle the entire process; I am capable of that*.” (P3). Furthermore, the interviews indicated that while EAS requests generally involve moral reflection, requests from patients with mental health conditions tend to raise additional ethical challenges. Some GPs questioned whether societal or healthcare shortcomings contributed to patients suffering. One GP reflected: “*So, elderly people who are lonely and never receive any visitors. Aren’t we, as a care system or society, failing them? The home care comes by twice a day, and they’re alone the rest of the time. The same goes for someone with a psychiatric illness who isn’t accepted enough.*” (P5). Others described experiencing personal discomfort: “*When it comes to psychiatric disorders or dementia, I find the ethical part difficult. It’s something I have to come to terms with personally. I’m also religious, and even though I do support euthanasia, I do so with certain boundaries.*” (P4).

#### Difficulties empathising with requests from patients with mental health conditions

GPs stressed that beyond assessing legal criteria, it is important for them to be able to empathise with a patient’s request. The interviews showed that this was not always straightforward in cases of mental health conditions. One GP explained: “*If someone was referred last year with a depression and now comes with that request, then I think, wait, that’s going very fast. But if I know someone who has been going from one psychiatric clinic to another for thirty years, and I know that he is utterly unhappy and has no one left, then that is much more understandable. For me, it is important whether it is something I can empathise with.*” (P2). Others emphasised that this capacity to empathise is central but can also be the greatest difficulty: “*It has to be something you can empathise with. And that really is possible with psychiatric patients. But I think that is where the greatest struggle lies.*” (P3).

Some GPs described a gap between recognizing suffering as real and being able to connect with it emotionally: “*I find psychiatric issues difficult to empathise with, even though I know it’s real and untreatable.*” (P5). In other cases, difficulties were linked to how patients relate emotionally, as one GP reflected: “*Look, there are people who are autistic and do not function on an emotional level in the same way as we do. With such a person, it is very difficult for me to connect on an emotional level, which makes it harder to feel at ease with something so important. That is, I think, something that cannot be captured in rules about euthanasia. But it has to be something you can empathise with.*” (P3). These accounts illustrate that GPs distinguish between understanding suffering at a cognitive level—acknowledging it as genuine and untreatable—and empathising with it at an emotional level.

#### Time, collaboration, and age considerations

Despite its complexities, many GPs believed requests related to mental health conditions can be responsibly assessed, but most said this requires more time and collaboration: “*And there are situations where it’s possible to say, 'Well, we’ve really tried everything. No one else thinks differently about this.' And we draw a line now, and we can indeed proceed with euthanasia. But again, it takes more time, it requires more consultation*.” (P13). For some, the extra time and complexity were reasons not to engage with such cases. Others stressed that time constraints should not interfere. Given the added difficulty, referrals to the ECE were more commonly discussed for requests related to mental health conditions than for those related to physical health conditions: “*I also had a few people with mental health issues. I couldn’t do it myself or didn’t want to, so I referred them to the Expertise Center Euthanasia* (P7) and “*I once had a young woman who requested it from me, but she consistently declined psychiatric care and wasn’t clearly suicidal either. I referred a case like that to the Expertise Center Euthanasia*” (P4).

For GPs who considered evaluating mental health requests or had done so in the past, patient characteristics—especially age—played an important role. Requests from younger patients were often met with hesitation. Some GPs felt uncomfortable deciding on such irreversible matters for younger individuals. One GP shared: “*I do have difficulty with euthanasia in cases of psychological suffering, especially in young people, because I wonder if someone's brain has fully developed.*” (P8). Another said: “*That was also the case with psychological suffering in patients of mine from practice. One was a 43-year-old woman and the other was a 65-year-old woman. I felt that the 43-year-old woman was really too young, so for myself I could not justify doing that.*” (P6).

#### The role of legal and professional frameworks

Lastly, GPs expressed appreciation for the existing legal framework and professional guidelines that help navigate complex euthanasia cases. Several emphasised that Dutch euthanasia law is well established and provides a clear foundation. As one GP stated: “*In the Netherlands, we have had a good law for quite some time now, and we are trained accordingly. So yes, as general practitioners, we know what we need to do, so to speak. We have good guidelines for that*.” (P10). Another remarked: “*I think the laws and regulations are clear about what is and isn't acceptable.*” (P11). Most GPs said they relied primarily on the KNMG guidelines: “*The rules we follow are from the KNMG. And indeed, the Dutch Association for Psychiatry also has separate guidelines. But as general practitioners, we follow the KNMG guidelines.*” (P6). However, most were unfamiliar with the Dutch Association for Psychiatry’s guidelines. SCEN-trained GPs or those with more experience knew of the psychiatric guidelines but sometimes found them too strict: “*What I find difficult is that the Dutch Association for Psychiatry imposes additional requirements. For example, the SCEN physician must also be a psychiatrist.*” (P1). Some GPs mentioned legal concerns specifically in cases involving mental health conditions: “*I find psychiatry more difficult because it maybe that even if you have done it correctly, you may have to stand in court.*” (P7). Still, most did not cite fear of prosecution as a major barrier when evaluating such requests.

## Discussion

### Study results in the context of existing evidence

This study provides new insights into Dutch general practitioners’ (GPs) willingness to grant or to perform euthanasia and assisted suicide (EAS) requests, with a comparison between patients suffering from mental health and physical health conditions. By combining quantitative survey data with qualitative interviews, the study not only confirms differences in willingness but also sheds light on the underlying reasoning and ethical considerations shaping GPs’ decisions.

Quantitative results showed that requests resulting from mental health conditions were significantly less likely to be granted compared to physical health conditions, and when asked directly, GPs were also less willing to perform them. This aligns with findings by Bolt et al., who reported that Dutch physicians found EAS more conceivable for physical health conditions (82%) than for mental health conditions (34%) [16]. The interviews help explain this discrepancy by illustrating why mental health suffering is experienced as more difficult to assess. GPs described challenges such as fluctuating symptoms, doubts about whether the wish to die stemmed from illness or a well-considered choice, difficulty assessing voluntariness, judging treatment exhaustion, and the possibility of recovery. These challenges, though not often compared directly with physical health cases, are well-documented in literature about EAS for patients with mental health conditions [13, 14, 18, 30].

At the same time, the interviews also revealed additional factors that have received less attention in previous studies. Several GPs expressed greater personal resistance to requests from younger patients with mental health suffering, suggesting that age plays a more decisive role in these cases. This aligns with findings from a Psychiatry study reporting concerns about future recovery, developmental maturity, and long-term prognosis in younger patients with mental health conditions [31]. Under Dutch law, additional safeguards apply when euthanasia is requested by minors. A minor must be deemed capable of making a reasonable appraisal of their interests. For patients aged 12–16, parental consent is required, while for those aged 16–17, parents must be consulted but their consent is not mandatory. The Euthanasia Code further highlights that in cases involving minors, as well as patients with mental health conditions, particular challenges can arise in determining whether a request is truly voluntary and well-considered [1, 5]. In general, establishing voluntariness and a well-considered request is already more difficult in younger patients, as decisional maturity and stability of wishes are harder to evaluate [5]. The combination of young age and mental health conditions may therefore explain why GPs are especially hesitant to perform euthanasia in such cases. Another important insight was that many GPs described feeling more emotionally distant from patients with mental health conditions, which made it harder for them to connect with and empathise with these requests. This emotional gap, often overlooked in previous research, may play a crucial role in explaining why willingness was lower in such cases as well.

In addition to these challenges, many GPs also admitted in the interviews that they feel less confident about their own competence in assessing cases from patients with mental health conditions and emphasised the need for psychiatric expertise to support their evaluations. This lack of confidence was mirrored in the survey results (Table 5), which showed that GPs were considerably less secure when assessing requests based on mental health conditions, aligning with previous studies [16, 18, 32]. Reflecting this uncertainty, several GPs reported that they were more likely to refer such patients to the ECE, where specialized teams could reassess the case, aligning with national data from the ECE [33]. This reliance on external expertise underscores both the perceived complexity of mental health conditions and the limits of GP confidence when navigating these requests.

These reflections carry important ethical implications. Lower willingness to grant requests from patients with mental health conditions does not necessarily indicate a lack of respect for autonomy, but rather a cautious response to the uncertainties of applying the due care criteria in this context. Similar concerns have been reported in prior qualitative studies, where GPs described struggling to balance protecting vulnerable patients with respecting their self-determination [15, 22]. Ten Cate et al. (2017) showed that, beyond the legal criteria, GPs’ judgments are also influenced by their own moral values [19]. The interviews in this study highlighted that besides the moral reflection each euthanasia request brings, requests from patients with mental health conditions raised additional ethical dilemmas. These included concerns about the broader social impact of granting such requests and whether doing so might lower the threshold for accessing the option to die, which may help explain the observed difference between physical and mental health cases as well.

Besides the disparity between GPs’ willingness toward granting requests based on mental health conditions and physical health conditions, this study showed that religion was another important factor influencing willingness. Religious GPs were less likely to grant EAS requests, consistent with prior findings that religious beliefs can act as a barrier [15, 17, 34]. Interestingly, religion did not significantly affect willingness to perform EAS. This suggests that granting and performing reflect different aspects of decision-making. Although in Dutch practice a GP unwilling to grant a request would also not perform it, this study deliberately distinguished between granting and performing in order to capture different dimensions of willingness. Religion was strongly associated with granting but not with performing, indicating that convictions may shape the formal assessment of requests more than the practical act of carrying them out.

Experience was another important factor. Quantitative results showed that prior experience performing EAS for patients with mental health conditions significantly increased willingness to do so again, consistent with research suggesting that direct experience reduces hesitation and builds confidence [34]. However, unlike Evenblij et al., who found that simply receiving requests from patients suffering from mental health conditions increased openness to performing them [13], this study observed no such effect. A likely explanation is that Evenblij et al. asked broader, more general questions about conceivability and included a wider range of physicians, whereas the present study focused solely on GPs and examined willingness more specifically. The interviews supported these findings: although almost all GPs had at some point received requests from patients with mental health conditions, many still felt unable or unwilling to perform them, emphasising that receiving a request alone does not provide the knowledge, confidence, or moral certainty needed to act. Some pointed to their lack of psychiatric expertise and the difficulty of distinguishing between illness-driven wishes and autonomous choices, while others described the heavier emotional burden and fear of acting prematurely. Interestingly, even GPs who had previously performed euthanasia for patients with mental health conditions acknowledged that such cases remain more difficult than physical ones, indicating that while experience performing euthanasia for patients suffering mentally may reduce hesitation, it does not resolve the fundamental uncertainties and ethical challenges associated with mental health conditions.

A further key finding was that euthanasia was more acceptable than assisted suicide, as shown in Table 5. This contrasts with a previous study that reported no significant difference between the two practices [35]. One explanation may lie in sample composition: the earlier study included elderly care physicians and specialists, while the present study focused solely on GPs. Since GPs receive and perform the majority of EAS requests in the Netherlands, they have much more experience with euthanasia than with assisted suicide. This greater familiarity may amplify the contrast between the two procedures, a pattern also highlighted in the interviews. GPs described euthanasia as more frequently encountered in practice, which reduced hesitation, while assisted suicide was less familiar and therefore approached with greater caution, consistent with findings from the fourth evaluation [17]. In addition, GPs emphasised that euthanasia allowed them to retain control over the process, whereas assisted suicide was perceived as less predictable and riskier. Together, these results suggest that both greater experience with euthanasia and a professional preference for control shape GPs’ stronger inclination toward euthanasia over assisted suicide.

Furthermore, although Table 5 showed that most GPs agreed or strongly agreed with the statement that “everyone has the right to decide over their own life and death,” the interviews revealed that putting this attitude into practice is far from easy. Even when the legal criteria were met, GPs described the decision to perform euthanasia as demanding, emotionally taxing, and morally weighty. They emphasised the importance of a strong doctor–patient relationship, moral reflection, and the emotional and organisational burden that accompanies euthanasia, confirming earlier studies [21, 22, 36]. And while earlier studies emphasised the importance of understanding patient requests, this study notably found that GPs highlighted the need to empathise with a patient’s wish, a dimension rarely discussed in existing literature [22]. Additionally, while some studies suggest that family opinions can significantly influence or even pressure physicians, most participants in this study valued family input without feeling pressured. This aligns with recent findings showing that Dutch physicians often seek relatives’ views, but only 35% ultimately consider them in their decisions [20, 21, 37].

Lastly, while most GPs reported using the KNMG guidance, our interviews showed limited familiarity with the NVvP guideline for EAS in case of mental health conditions. Table 5 indicates general dissatisfaction with the current guidelines used to assess mental-health cases. Taken together, these findings suggest that dissatisfaction may be driven by low awareness and limited uptake of existing resources. Although the NVvP guideline is not formally required by the RTE, better implementation—for example, making the guidance easier to use in GP practice and providing training on its application—could increase awareness and reduce dissatisfaction. In turn, this may support more consistent decision-making and reduce variation in how requests from patients with mental health conditions are assessed.

### Strengths and limitations

This study used a concurrent mixed-methods approach, combining quantitative and qualitative data to strengthen validity. While the survey identified broad patterns in GPs willingness toward EAS, interviews provided deeper insight into the reasoning behind their decisions, enhancing the study’s comprehensiveness and interpretative depth. The research is particularly timely given the rise in EAS cases in the Netherlands and the growing international debate surrounding such practices. Insights from the Dutch context may serve as a useful reference for countries navigating similar legal and ethical considerations [4, 24].

Nevertheless, this study has some limitations. The relatively small sample size (n = 103) may limit the generalizability of the findings, although most results are in line with prior research. Some GPs indicated that the survey answer options did not fully reflect their views, particularly regarding the lack of an option to refer a patient’s request. Furthermore, due to convergence issues, multilevel modelling was not used for Models 4 and 5. Instead, binary logistic regression without hierarchical structure was applied. Odds ratios (ORs) and confidence intervals (CIs) for most predictors remained consistent with those in the hierarchical models, indicating that this modelling approach did not impact the interpretation of the results. There were also some missing values in the survey data, which could have introduced bias if not addressed. To minimize this risk, variables with a large proportion of missing responses (such as gender) were excluded from the final models, while variables with only a small number of missing responses (e.g., practice area) were retained and handled through case wise deletion. This approach ensured that the effective sample size remained robust and that the overall validity of the findings was not compromised.

The qualitative strand also has some limitations. Interviews were both carried out and coded by the first author. While this may raise the risk of bias, it is mitigated by the fact that the first author has substantial training in qualitative research methods, experience in interviewing on sensitive topics, and used a structured interview guide that was reviewed by the second author. In addition, although some interviews were conducted online or by telephone rather than face-to-face, this was largely due to geographical distance between interviewer and participants, and participants appeared comfortable sharing their views in this format. A further limitation concerns the use of hypothetical case examples in the survey. While it may have been more difficult for GPs to empathise with a case example than with a real-life case, the scenarios were carefully developed based on validated questionnaires from the fourth evaluation and earlier studies such as Kouwenhoven et al., which increases their credibility and comparability. Finally, it cannot be ruled out that some respondents may have worked in the same GP practice. Because no practice identifiers were collected in order to safeguard anonymity, clustering effects could not be controlled for. However, given the distribution of GPs across practices in the Netherlands, it is unlikely that a substantial proportion of respondents in this study came from the same practice. Therefore, this is not expected to have had a major impact on the results.

### Recommendations for future research

As EAS requests from patients with mental health conditions rise in the Netherlands, ongoing research remains critical. This study highlights the need to explore empathy in greater depth, particularly how it affects physicians’ willingness to engage with requests from individuals suffering from mental health conditions. Future qualitative research should further examine how patient characteristics, especially younger age, shape GPs’ decisions, as youth often provokes stronger ethical hesitation. Additionally, larger-scale quantitative studies are recommended to assess how physician traits such as experience, religion, and exposure to EAS requests related to mental health conditions influence clinical decisions. For future research, it would also be valuable to examine how GPs interpret and apply the Euthanasia Code and the ‘extra’ requirements for mental health cases as formulated by the RTE, since this did not explicitly emerge in our interviews but may substantially shape practice. In addition, future studies should further investigate how GPs’ willingness to grant requests from patients with mental health conditions is influenced by the presence of physical comorbidities, as such combinations frequently occur in practice and may substantially shape physicians’ judgments. These insights can inform more tailored training, support structures, and policies to better equip GPs for complex EAS requests.

## Conclusion

Dutch GPs are less willing to grant and perform euthanasia and assisted suicide requests from patients with mental health conditions than from those with physical health conditions. This reflects difficulties in assessing unbearable and irremediable suffering, clinical uncertainty, instability or illness-related influence on the request being well-considered, and challenges in empathising with patients’ experiences. Such requests are typically more prolonged, ethically demanding, and reliant on psychiatric input, with younger age and personal comfort adding further hesitation. Many GPs also reported limited awareness of existing guidelines for mental health–related requests. These findings underline the need to strengthen the implementation of guidance in GP practice, ensure timely psychiatric expertise and SCEN consultations, enhance collaboration with the ECE, and provide targeted training and practical tools to support consistent application of the due care criteria [38].

## Supplementary Information

Additional file 1.

Additional file 2.

Additional file 3.

## Data Availability

The datasets used and/or analyzed during the current study are available from the corresponding author on reasonable request.

## Abbreviations

CI: Confidence interval
EAS: Euthanasia and assisted suicide
ECE: Expertise center euthanasia
GP: General practitioner
KNMG: Royal Dutch medical association
MAXQDA: Software for qualitative and mixed methods data analysis
NVvP: Dutch association of psychiatrists (Nederlandse Vereniging voor Psychiatrie)
OR: Odds ratio
RTE: Regional euthanasia review committees
SCEN: Support and consultation on euthanasia in the Netherlands
SPSS: Statistical package for the social sciences
