# Supplementary material for: Willingness of Dutch general practitioners to grant euthanasia and assisted suicide requests: a comparative study of physical and mental health conditions
Source: BMC Med Ethics. 2025 Nov 25;26:179. doi: 10.1186/s12910-025-01333-y (PMC12752032; doi:10.1186/s12910-025-01333-y)
Supplement: Supplementary file 1 — Additional file 1. [file 12910_2025_1333_MOESM1_ESM.docx]

**Section 1: Experience with EAS Requests**

1. Have you ever received a direct request for EAS from a patient with a somatic or psychiatric condition?
   - Yes
   - No
   - No answer
2. Has a patient with a somatic condition ever explicitly requested EAS from you (i.e., the request is solely based on somatic suffering)?
   - Yes
   - No
   - No answer
3. Have you ever performed EAS for a patient whose request was solely based on somatic health problems?
   - Yes
   - No
   - No answer
4. Has a patient with a psychiatric condition ever explicitly requested EAS from you (i.e., the request is solely based on psychiatric suffering)?
   - Yes
   - No
   - No answer
5. Have you ever performed EAS for a patient whose request was solely based on psychiatric health problems?
   - Yes
   - No
   - No answer

**Section 2: Case examples**

**Instruction:** For each of the following case examples, please indicate whether you would grant the request.

*(Each case is presented in two formats: one requesting euthanasia, the other requesting assisted suicide)*

**Case 1 – ALS (Amyotrophic Lateral Sclerosis): Mr. Van de Berg**

**Euthanasia request:**
Mr. Van de Berg suffers from ALS. He has lost nearly all motor functions, including the ability to walk, speak, and swallow independently. Despite receiving supportive care, he is completely dependent on others for all daily activities. His condition will inevitably lead to respiratory failure. He finds his current and future situation unbearable and submits a request for euthanasia.

**Assisted suicide request:**
Mr. Van de Berg suffers from ALS. He has lost nearly all motor functions, including the ability to walk, speak, and swallow independently. Despite supportive care, he is entirely dependent on others for daily life. His condition will inevitably lead to respiratory failure. He finds his current and future situation unbearable and asks for a life-ending drug that he can self-administer.

**Case 2 – Terminal Breast Cancer: Ms. De Jong**

**Euthanasia request:**
Ms. De Jong has metastatic breast cancer. She has undergone several treatments, but her disease is no longer curable. She experiences severe, hard-to-manage pain and feels she is losing control over her life, a sense of control she valued deeply during her working life. She states she cannot go on like this and requests euthanasia.

**Assisted suicide request:**
Ms. De Jong has metastatic breast cancer. She has undergone several treatments, but her disease is no longer curable. She experiences severe, hard-to-manage pain and feels she is losing control over her life, a sense of control she valued deeply during her working life. She says she can no longer cope and asks her GP for a life-ending drug that she can take herself.

**Case 3 – Chronic PTSD: Mr. Dijkstra**

**Euthanasia request:**
Mr. Dijkstra has experienced severe trauma in the past, resulting in chronic PTSD. He suffers from constant flashbacks, nightmares, and emotional distress that have not improved despite years of intensive treatment. Mr. Dijkstra requests euthanasia, and a second independent physician confirms that he meets all legal criteria.

**Assisted suicide request:**
Mr. Dijkstra has a history of severe trauma, leading to chronic PTSD. He lives with persistent flashbacks, nightmares, and emotional distress that have not improved despite years of intensive treatment. Mr. Dijkstra asks his GP for a life-ending drug that he can self-administer, and a second independent physician confirms that all legal criteria are met.

**Case 4 – Schizophrenia: Mr. Jansen**

**Euthanasia request:**
Mr. Jansen has lived with schizophrenia for many years. Despite consistent treatment with medication and therapy, he continues to experience severe hallucinations and delusions that significantly impair his quality of life. These symptoms cause him immense suffering, and he sees no hope for improvement. After careful consideration, he requests euthanasia from his GP.

**Assisted suicide request:**
Mr. Jansen has lived with schizophrenia for many years. Despite consistent treatment with medication and therapy, he continues to experience severe hallucinations and delusions that significantly impair his quality of life. These symptoms cause him immense suffering, and he sees no hope for improvement. After careful consideration, he requests his GP for a life-ending drug he can take himself.

**Case 5 – Treatment-resistant Depression: Ms. Langezaal**

**Euthanasia request:**
Ms. Langezaal is physically healthy but suffers from severe, long-term depression. Psychiatric treatments have failed to relieve her symptoms. She frequently tells her doctors she wants to die and has previously attempted suicide, unsuccessfully. She asks her GP for euthanasia to end her suffering.

**Assisted suicide request:**
Ms. Langezaal is physically healthy but suffers from severe, long-term depression. Psychiatric treatments have failed to relieve her symptoms. She frequently tells her doctors she wants to die and has previously attempted suicide, unsuccessfully. She asks for a life-ending drug that she can take herself.

**Case 6 – Severe Autism Spectrum Disorder: Ms. Smit**

**Euthanasia request:**
Ms. Smit has a severe form of Autism Spectrum Disorder (ASD), which has caused lifelong suffering. She experiences constant sensory overload, extreme social isolation, and relentless psychological pain. Despite decades of therapy and medication, nothing has relieved her suffering. She is unable to form relationships, participate in daily life, or find relief from the overwhelming stimuli she faces. She requests euthanasia.

**Assisted suicide request:**
Ms. Smit has a severe form of Autism Spectrum Disorder (ASD), which has caused lifelong suffering. She experiences constant sensory overload, extreme social isolation, and relentless psychological pain. Despite decades of therapy and medication, nothing has relieved her suffering. She is unable to form relationships, participate in daily life, or find relief from the overwhelming stimuli she faces. She asks for a life-ending drug she can take herself to end her suffering.

**Section 3: Attitudes Toward EAS**

**Instructions:** Please indicate your level of agreement using the Likert scale below:
1 = Strongly disagree | 2 = Disagree | 3 = Neutral | 4 = Agree | 5 = Strongly agree | No answer

1. Everyone has the right to decide about their own life and death.
2. Patients with somatic conditions should be eligible for EAS (upon request).
3. Patients with psychiatric conditions should be eligible for EAS (upon request).
4. Psychiatric patients should have the same right to request EAS as somatic patients.
5. I feel less confident assessing EAS requests from psychiatric patients than from somatic patients.
6. It is impossible to determine whether a psychiatric patient suffers unbearably and without prospect of improvement.
7. It is impossible to assess whether a psychiatric patient’s death wish is well-considered.
8. It is impossible to assess whether a psychiatric patient’s death wish stems from psychopathology.
9. The current guidelines provide sufficient clarity for evaluating EAS requests from psychiatric patients.
10. I would be more willing to provide assisted suicide than euthanasia for psychiatric patients.
11. I would be more willing to provide assisted suicide than euthanasia for somatic patients.

**Section 4: Willingness to Perform EAS**

1. **I am willing to perform EAS for a patient with a somatic condition.**
   - Agree
   - Disagree
   - No answer
2. **I am willing to perform EAS for a patient with a psychiatric condition.**
   - Agree
   - Disagree
   - No answer

**Section 5: Demographics**

1. **What is your gender?**
   - Female
   - Male
   - No answer
2. **What is your age?**
   - ≤40
   - 40–54
   - 55
   - No answer
3. **What is your ethnicity?**
   - Dutch
   - Non-Dutch
   - No answer
4. **What is your religious affiliation?**
   - Catholic
   - Protestant
   - Muslim
   - Other
   - None
   - No answer
5. **How many years have you worked as a physician?**
   - 2–5
   - 5–10
   - 10
   - No answer
6. **What type of area do you primarily work in?**
   - Urban
   - Rural
   - No answer
