# Supplementary material for: Willingness of Dutch general practitioners to grant euthanasia and assisted suicide requests: a comparative study of physical and mental health conditions
Source: BMC Med Ethics. 2025 Nov 25;26:179. doi: 10.1186/s12910-025-01333-y (PMC12752032; doi:10.1186/s12910-025-01333-y)
Supplement: Supplementary file 2 — Additional file 2. [file 12910_2025_1333_MOESM2_ESM.docx]

**General**

- Experience with EAS requests
- Perspectives on EAS for somatic vs. psychiatric patients

**Guidelines and Legal Framework**

- Familiarity with legal and professional guidelines (incl. NVvP)
- Clarity and usefulness of guidelines
- Uncertainty about due care criteria
- Legal concerns

**Decision-Making Process**

- Process for psychiatric vs. cases
- Key challenges in decision-making
- Difficult-to-assess due care criteria psychiatric vs. somatic
- Assessing treatment exhaustion
- External pressures (colleagues, family, society)

**Ethical Considerations**

- Moral and ethical dilemmas
- Differences between patient groups

**Additional Topics**

- Role of GPs in psychiatric EAS
- Confidence and knowledge in handling psychiatric vs. somatic cases

**Closing**

- Suggestions for improvement
- Final remarks or additions
