# Supplementary material for: Willingness of Dutch general practitioners to grant euthanasia and assisted suicide requests: a comparative study of physical and mental health conditions
Source: BMC Med Ethics. 2025 Nov 25;26:179. doi: 10.1186/s12910-025-01333-y (PMC12752032; doi:10.1186/s12910-025-01333-y)
Supplement: Supplementary file 3 — Additional file 3. [file 12910_2025_1333_MOESM3_ESM.docx]

| **Main code** | **Subcodes** | **Definition** | **Example from interview** | **Encoding rule** |
| --- | --- | --- | --- | --- |
| Assessment confidence | - Somatic - Psychiatric | Physicians’ confidence in assessing patients requests for EAS | *"Both my colleague and I hesitated for quite some time. Does she really want this?”* | Code this when physicians express how confident or uncertain they feel when assessing somatic and psychiatric cases |
| Decision-making capacity | - Somatic - Psychiatric | Evaluation of the patient’s decisional competence related to the EAS request. | *“And then I said: yes, then I’ll be there for you but only if you can clearly show that this is what you want, that it’s your choice”* | Apply when the physician discusses the patient’s mental capacity to make an informed decision. |
| Emotional burden |  | The emotional weight or toll physicians experience due to their involvement in EAS cases. | *“You have to do it very carefully. But it's also emotionally charged. Because with all the people for whom I’ve performed euthanasia, I developed a connection, we had many conversations, especially about why they wanted it”* | Use when the physician discusses the emotional toll or burden they face when handling an EAS requests |
| Empathy | - Somatic - Psychiatric | Emotional sensitivity or compassion expressed by the physician in EAS-related care. | *“Who also sees himself failing in his physical self-care. Who notices that he sometimes can’t find the toilet. And then you really experience the suffering caused by this mental disorder. That’s when it becomes very palpable”* | Tag when physicians express empathy or relate to the patients EAS requests |
| Ethical considerations | - Somatic - Psychiatric | Mentions of moral or ethical conflicts that arise in the context of EAS decision-making. | *“When it comes to psychiatric disorders or dementia, I find the ethical part difficult”* | Apply to passages where moral dilemmas or ethical considerations are mentioned. |
| Experience | - Somatic - Psychiatric | Reflections on professional experience with EAS requests. | *“So far, I’ve personally performed euthanasia once for a patient suffering psychologically, and I found it very complex”* | Use when physicians reflect on their practical experience with EAS requests. |
| Family involvement | - Somatic - Psychiatric | Involvement of family members in the process, including their influence on decisions. | *“Usually, the family cooperates, but they’re not quite ready yet”* | Code when the role of family members is mentioned within the EAS process |
| Guideline use | - Somatic - Psychiatric | Use and clarity of official guidelines relevant within the EAS decision-making process | *“* *So in that case, I find the guideline that the Association for Psychiatry wants to implement actually too strict”* | Apply when physicians refer to official guidelines or their clarity/usefulness. |
| Influence of patients characteristics | - Somatic - Psychiatric | How patient-specific traits affect the physician's decision. | *“* *And it’s not just because it’s psychiatry—if she were fifty, with her entire history, it would already be different. But someone that young... I couldn’t bring myself to end her life, because that’s what you’re doing”* | Code when physicians discuss patients characteristics that influenced their decision |
| Knowledge gap | - Somatic - Psychiatric | Lack of knowledge, uncertainty, or insufficient training in assessing or handling EAS cases. | *"And in that, I—at least—really need the expertise of a psychiatrist to help assess the situation. To understand: what stems from the illness, and what stems from the wish to die, the desire to no longer live this life? And what, if anything, is still treatable in that?”* | Apply when physicians state or indicate they lack knowledge to evaluate the EAS requests |
| Legal concerns | - Somatic - Psychiatric | Concerns about possible legal consequences or risks of participating in EAS. | *“I find psychiatry more difficult. Because it’s possible that, if you did it right, you still have to stand before a court”* | Use when physicians indicate they fear legal consequences if/when participating in EAS |
| No treatment options left | - Somatic - Psychiatric | Mentions of assessing whether all treatment options have been tried | *“What I find challenging is that determining whether something is still treatable or truly hopeless is often harder to objectify in psychiatry”* | Apply to comments regarding treatment exclusion |
| Pressure in decision making | - Somatic - Psychiatric | Feelings of external pressure from patients, families, colleagues, or societal expectations. | *And if someone is indeed in the final stage—like with a brain tumour—drowsy and no longer able to clearly express what they want, then you’ve basically passed the point where euthanasia is still possible. Once, under pressure from the family, I still called in the SCEN physician, but after speaking with the patient, they said: ‘This lady can no longer independently express what she wants. We cannot proceed with euthanasia in this case.* | Apply when external pressures (from family, colleagues, etc.) are described. |
| Preventive consultations |  | This refers to situations where patients ask about euthanasia even before they are in a critical or terminal stage of illness. | *“” People ask, sometimes when they are still very healthy, "Well, would you do that as a doctor?"* | Code when GP describes that a patient asked for EAS while not being seriously sick or terminally ill. |
| Process time | - Somatic - Psychiatric | Time-related factors in the EAS decision process, including prolonged procedures or evaluations. | *“To explore that thoroughly within a psychiatric case, you need an enormous amount of time”* | Apply when time-related aspects of the process are discussed (e.g. delays, length). |
| Professional boundaries | - Psychiatric - Somatic | Where physicians draw the line within the EAS process | *“When people ask about euthanasia and they’re not in the terminal stage of an illness—when the question is more like, ‘I'm in the early stages of dementia and I want euthanasia’—then I say no”* | Code any segment where the physicians discuss that they are not willing to cooperate within the process |
| Psychopathology Influence |  | Influence of a patient’s psychiatric condition making it harder to evaluate the EAS request | *“I currently have some psychiatric patients who have made a request. But what I find difficult is that I’m not a psychiatrist. So I can’t really judge whether that wish stems from a depression, a personality disorder, or from an inability to cope that’s part of their condition”* | Use when psychiatric symptoms influence the evaluation of the EAS request. |
| Referral behaviour | - Somatic - Psychiatric | Referring patients to expertise center or other physicians with their EAS requests | *“Also a few people with psychological problems. In those cases, I couldn’t or didn’t want to carry it out myself, so I referred them to the Expert Centre for Euthanasia”* | Code when physician mentions they referred a patient with an EAS request |
| Suffering assessment | - Somatic - Psychiatric | How unbearable suffering is assessed or interpretated | *“In other words, psychiatric suffering is more complex in that it's harder to determine compared to most somatic conditions”* | Use when suffering is assessed or described, |
| Support second physician |  | In general expressing needing a second physician for support within the EAS process | *So that does mean that the path you follow is, let’s say, less standardized, and that I would also end up asking more people around me in such a situation and would need to seek the expertise of others* | Use when need for second physician in either performing or evaluating in mentioned |
